# The neuroprogressive nature of major depressive disorder: evidence from an intrinsic connectome analysis

**DOI:** 10.1038/s41398-021-01227-8

**Published:** 2021-02-04

**Authors:** Jin Liu, Yiming Fan, Ling-Li Zeng, Bangshan Liu, Yumeng Ju, Mi Wang, Qiangli Dong, Xiaowen Lu, Jinrong Sun, Liang Zhang, Hua Guo, Futao Zhao, Weihui Li, Li Zhang, Zexuan Li, Mei Liao, Yan Zhang, Dewen Hu, Lingjiang Li

**Affiliations:** 1 Department of Psychiatry, The Second Xiangya Hospital, Central South University, Changsha, Hunan China; 2 Mental Health Institute of Central South University, China National Clinical Research Center on Mental Disorders (Xiangya), China National Technology Institute on Mental Disorders, Hunan Technology Institute of Psychiatry, Hunan Key Laboratory of Psychiatry and Mental Health, Changsha, Hunan China; 3 College of Intelligence Science and Technology, National University of Defense Technology, Changsha, Hunan China; 4 Zhumadian Psychiatric Hospital, Zhumadian, Henan China

**Keywords:** Depression, Prognostic markers, Molecular neuroscience

## Abstract

Major depressive disorder (MDD) is a prevailing chronic mental disorder with lifetime recurring episodes. Recurrent depression (RD) has been reported to be associated with greater severity of depression, higher relapse rate and prominent functioning impairments than first-episode depression (FED), suggesting the progressive nature of depression. However, there is still little evidence regarding brain functional connectome. In this study, 95 medication-free MDD patients (35 with FED and 60 with RD) and 111 matched healthy controls (HCs) underwent resting-state functional magnetic resonance imaging (fMRI) scanning. After six months of treatment with paroxetine, 56 patients achieved clinical remission and finished their second scan. Network-based statistics analysis was used to explore the changes in functional connectivity. The results revealed that, compared with HCs, patients with FED exhibited hypoconnectivity in the somatomotor, default mode and dorsal attention networks, and RD exhibited hyperconnectivity in the somatomotor, salience, executive control, default mode and dorsal attention networks, as well as within and between salience and executive control networks. Moreover, the disrupted components in patients with current MDD did not change significantly when the patients achieved remission after treatment, and sub-hyperconnectivity and sub-hypoconnectivity were still found in those with remitted RD. Additionally, the hypoconnectivity in FED and hyperconnectivity in RD were associated with the number of episodes and total illness duration. This study provides initial evidence supporting that impairment of intrinsic functional connectivity across the course of depression is a progressive process.

## Introduction

Major depressive disorder (MDD) is a clinical progressive mental disorder in nature. It was reported that up to 85% of MDD patients who have achieved remission would develop at least one new episode in the next 15 years^1^. According to some studies, the number of episodes was positively correlated with the severity of symptoms, duration, vulnerability to developing new episodes, and risks of recurrence^2–4^. The World Health Organization has listed MDD as a major cause of disability because its progressive nature is associated with poor prognosis, loss of working ability, impaired social function and high disease burden^5–8^.

Many studies support that abnormal brain structural and functional alterations are the underlying pathophysiology of MDD^9–11^. Previous studies consistently reported the associations between brain structure alterations and the course of illness (e.g., the number of episodes and the illness duration). Negative correlations were found between a greater number of prior depressive episodes and a reduction in the hippocampal and amygdala volume^12,13^, as well as the thinning of medial prefrontal cortex (mPFC)^14^. It was also found that illness duration was correlated with the volume reduction of hippocampus, putamen, insula and mPFC^15–17^. Nonetheless, only a small number of studies have examined the relationship between the course of illness and brain functional alterations prospectively to explain the progressive nature of depression.

Abnormal interactions across the default mode (DMN), executive control (ECN), salience (SN), limbic (Limbic), dorsal attention (DAN) and somatomotor (SMN) networks can lead to a wide range of affective, cognitive and somatic symptoms in patients with MDD^9,18^. Cross-sectional studies reported that the functional connectivity (FC) alterations of the right putamen network, precuneus and the hypoconnectivity of the left posterior cingulate cortex to DMN, and the hypoconnectivity of the amygdala to a large part of the SN were closely related to the number of episodes^19–21^. Greicius et al. found that the abnormal FC in the subgenual cingulate was associated with the duration of a depressive episode^22^. Additionally, it was suggested that altered FC between amygdala and subgenual anterior cingulate cortex was associated with future relapse^23^. These findings support the view that depression is related to the FC impairment over a progressive course of illness. However, the associations between the course of illness and the brain functional alterations is only indirect evidence for the progressive nature of depression.

To confirm the progressive nature of brain functional alteration in MDD, longitudinal studies repeatedly measuring the brain functional alterations in each progressive episode of the same MDD patients are essential. However, few studies adopted such a design. Previous researches have revealed varying extent of FC aberrance across different functional connectivity networks (FCNs) in patients with FED and those with RD^24–28^. In a study by Yan et al.^29^, it was found that FED patients only showed decreased connectivity within visual network (VN), while RD patients showed significantly decreased intra-network connectivity of VN, SMN, and DMN, and decreased inter-network connectivity of VN-SMN, VN-DAN, and SMN-DAN. And this study also suggested that RD was associated with more severe FC disruption and more extensive FCN abnormality than FED, indicating that the number of episodes might be related to brain functional alterations. Considering that this study did not show an obvious correlation between FC aberrance and illness, evidence is still insufficient to identify the progression of FC abnormalities in patients with MDD.

To identify the progressive nature of intrinsic FC in a dynamic disease course, we constructed large-scale intrinsic FCNs in 95 medication-free MDD patients and 111 matched healthy controls (HC) across a 6-month period. Network-Based Statistic (NBS) analysis was used to analyze the intrinsic FC aberrance in the patients with FED and RD in both the episode phase and the remission phase. The correlation between intrinsic FC and the number of episodes as well as the total illness duration was also analyzed. Our hypothesis is that MDD is a progressive disease with more extensive and prominent FC aberrance in RD than FED. Additionally, some aberrant connections might remain abnormal even in the remission phase and might be correlated with the number of episodes and total illness duration.

## Materials and methods

### Participants

One hundred and seven patients with MDD and 117 healthy controls (HC) were recruited from Zhumadian Psychiatric Hospital (Henan, China) and its surrounding communities from 2013 to 2018(chictr.org, ChiCTR1800014591). The inclusion and exclusion criteria of the two groups were detailed in Supplementary Information. This study was approved by the Medical Ethics Committees of the Second Xiangya Hospital of Central South University and the Zhumadian Psychiatric Hospital. Written informed consent forms were obtained from all the participants.

### Treatment and efficacy assessment

All the subjects underwent the fMRI scanning at baseline. The patients with MDD received paroxetine for 6 months based on the judgment of their physician. Their dosage started at 10 mg daily, and was increased to 20 mg or higher in the second week. The maximum dosage was 60 mg daily, based on the severity of symptoms, clinical responses and side effects. After baseline assessment and scanning, the MDD patients were assessed using HAM-D_24_ at the end of the 0.5, 1^st^, 2^nd^, 3^rd^, 4^th^, 5^th^ and 6^th^ months. At the end of the 6^th^ month, the patients received other clinical assessments and a second MRI scan. And the patients with a HAM-D_24_ score of ≤ 7 for at least two months was regarded as clinical remission. Of the 107 patients enrolled, 5 had excessive head motions (more than 2 mm of translation and 2° of rotation in any of the *x*-, *y*- and *z*-axes), and 7 experienced manic episodes during the six months of treatment; thus, their data were removed from the study. A total of 95 patients (35 with FED and 60 with RD) were included in further analyses. During the treatment, 7 patients received electroconvulsive therapy or other antidepressant agents, and 25 patients discontinued their participation. Thus, a total of 63 patients finished the 6-month treatment period and underwent the second MRI scan, with 56 clinically remitted patients (20 with FED and 36 with RD) (3 RD patients had a new episode during treatment and achieved clinical remission at the end of the 6^th^ month) included in the final analyses while 7 unremitted patients excluded due to the relatively small number of them.

A group of 117 matched HCs also underwent the baseline scan and clinical assessments, with 6 excluded for excessive head motions.

### fMRI data acquisition, preprocessing and FC networks construction

All the subjects were scanned using a 3 T MR scanner (Signa HDxt MR, GE Healthcare). A preprocessing approach similar to that in our previous studies was used^30,31^. Details of MRI data acquisition and preprocessing are described in the Supplementary Information.

To generate the whole-brain functional connectome, we used a previously established functional parcellation of the cerebral cortex and striatum to decompose the whole brain FC into 7 resting-state networks, namely VN, SMN, Limbic, ECN, DAN, SN and DMN (Supplementary Fig. S1)^32,33^. Across all the 7 sub-networks, there are 132 separated anatomical regions of interest (ROIs), which were then used to represent nodes in FC networks. The functional connection between any two nodes *i* and *j* was defined as the Fisher-z transformed Pearson product-moment correlation of the averaged blood oxygen level-dependent (BOLD) time courses within these regions. The averaged BOLD time series of an ROI was obtained by averaging the time series of all the voxels in this ROI. The Pearson’s correlation coefficients were then calculated between each pair of ROIs. To improve the normality of the correlation coefficients, Fisher’s r-to-z transformation was performed to convert the correlation coefficients to z-values (see Supplementary Information).

### Network-based statistics (NBS) analysis

NBS analysis was performed to identify any connected components that were significant in a set of altered connections found in patients MDD compared to HCs^34^. NBS analysis was implemented in four steps: firstly, the hypothesis of interest at every connection was statistically tested independently; secondly, a statistical threshold was set as the primary threshold; and then, topological clusters were identify among the set of supra-threshold connections using a breadth search; finally, an FWER-corrected *p*-value for each component was calculated using permutation testing (Supplementary Information).

The first three steps of the NBS analysis were repeated for the data of each permutation. In particular, the hypothesis of interest was tested for every connection using the same statistical test. With a set of supra-threshold connections defined using the same threshold, any connected graph components were then identified. The size of the largest component was recorded for each permutation, thereby yielding an empirical null distribution for the size of the largest component. Finally, the one-sided FWER-corrected *p*-value of a given-sized component was then estimated as the percentage of the largest components during permutations against the total number of permutations.

### Statistical analysis

The specific implementation of NBS analysis consisted of two steps. Firstly, the significantly abnormal components across FED, RD, and HC groups were identified using the analysis of covariances (ANCOVA). And then, the NBS analyses of FED vs. HC, RD vs. HC, and FED vs. RD were performed, with the abnormal components as the connection masks. Age, gender, education, and mean frame-wise displacement (FD) were controlled as covariates in all the four comparisons. The number of permutations was 5,000 and the statistical threshold was set at *t* = 3.0. The significant level of FWER-corrected *p*-value was set at 0.001. The significant components were displayed in BrainNet Viewer^35^.

To investigate the alteration of the disrupted components in the patients with FED or RD between the episode and the remission phase, we performed NBS analysis for remitted FED (rFED) vs. rFED-pretreatment, remitted RD (rRD) vs. rRD-pretreatment, rFED vs. HC, and rRD vs. HC by using the disrupted components of the FED and RD groups as masks, respectively. The same data preprocessing and FCN construction were performed on remitted MDD (rMDD) patients. The NBS analysis between the two subgroups in the episode phase was also performed to determine that the 56 pre-treatment rMDD patients was comparable with the 39 dropouts and unremitted patients in terms of FC.

Pearson correlation analyses were conducted to assess any significantly linear associations between clinical variables and the average FC values of abnormal components of the FED and RD groups, with *p* < 0.05 being statistically significant.

## Results

### Demographic and clinical characteristics

The detailed demographic and clinical characteristics of the MDD (FED and RD), rMDD (rFED and rRD) and HC groups were presented in Supplementary Table S1. There was no significant difference regarding age, gender, and education level between the FED, RD, and HC groups, and between the rFED, rRD and HC groups. Additionally, there was no significant difference in the HAM-D_24_ total score between the FED and RD groups, and between the rFED and rRD groups.

### Abnormal FC in FED and RD

A significant disrupted component consisting of 48 connections (*p* < 0.05, the primary threshold *F* = 6) was found in the FED and RD groups, compared with the HCs (Supplementary Fig. S2). Specifically, the component mainly included inter-network connectivity of SMN-SN, SMN-DAN, SMN-DMN, SMN-ECN, SN-ECN, DAN-ECN, and intra-network connectivity of SN and ECN (Supplementary Table S2).

**Fig. 1:**
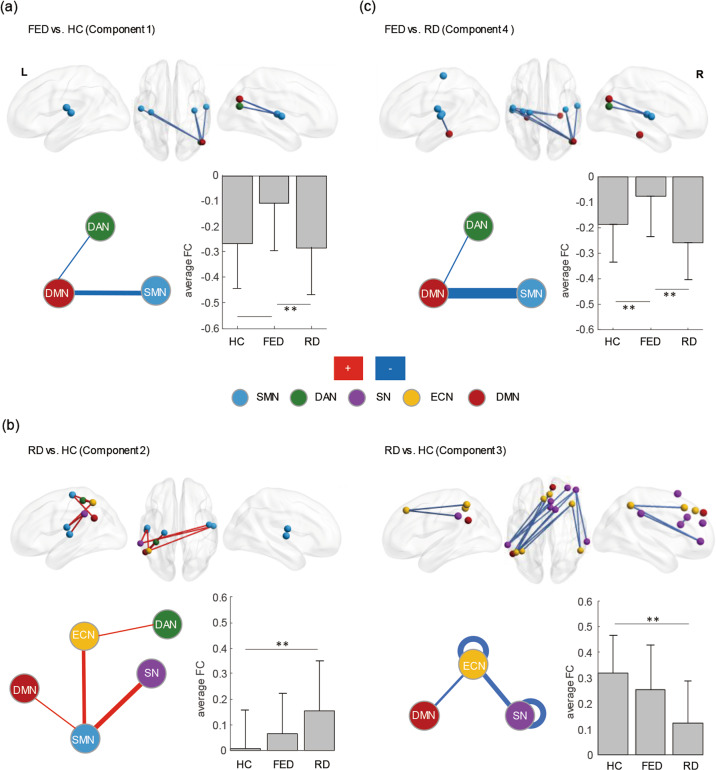
Disrupted components in FED and RD. The components were identified using the NBS analysis (the top row). The colors of nodes indicate their intrinsic functional connectivity network (FCN) membership as defined by the Yeo parcellation (the bottom row). The thickness of the lines represents the number of significant intra- (loops) and inter-network connections, with thicker lines representing a greater number of significant connections. The blue lines represent hypoconnectivity in patients with FED or RD. The red lines represent hyperconnectivity in patients with RD, compared to HCs. **p < 0.001, *p < 0.05. **a** Hypoconnectivity in patients with FED, compared to HCs (Component 1). **b** Hyperconnectivity and hypoconnectivity in patients with RD, compared to HCs (Component 2 and Component 3, respectively). **c** Hypoconnectivity in patients with FED, compared to RD (Component 4). SMN: somatomotor network, DAN: dorsal attention network, SN: salience network, ECN: executive control network, DMN: default mode network, L: left, R: right, FED: first-episode depression, RD: recurrent depression, HC: healthy control.

A disrupted component with hypoconnectivity was found in the FED group, compared to HCs (*p* < 0.001; Fig. 1a and Table 1). The component (Component 1, including 5 connections) mainly included inter-network connectivity of SMN-DMN. Besides, the average FC value of hypoconnectivity in the FED group was significantly lower than those with RD and the HCs, while no significant difference was found between the RD group and the HCs.

**Table 1:** Four disrupted components and aberrant functional connectivity in FED or RD identified using network-based statistical analysis.

Significant connections					Functional connectivity		
Network	Seed region	Coordinates (*x*, *y*, *z*)	Target region	Coordinates (*x*, *y*, *z*)	FED	RD	HC
*FED vs. HC (Component 1)*							
SMN-DAN	R—Insula	(36, −18, 9)	R – Parieto-occipital cortex	(46, −70, 20)	0	−0.17	−0.15
SMN-DMN (4)	L—S2	(−50, −15, 17)	R—Inferior parietal lobule	(49, −70, 30)	−0.18	−0.36	−0.32
	L—Insula	(−34, −21, 10)	R—Inferior parietal lobule	(49, −70, 30)	−0.06	−0.21	−0.19
	R—Insula	(36, −18, 9)	R—Inferior parietal lobule	(49, −70, 30)	−0.06	−0.23	−0.23
	R—Auditory cortex	(55, −12, 6)	R—Inferior parietal lobule	(49, −70, 30)	−0.26	−0.44	−0.44
*RD vs. HC (Component 2)*							
SMN-SN (4)	L—S2	(−50, −15, 17)	L—Inferior parietal lobule	(−61, −40, 36)	0.21	0.28	0.12
	L—Auditory cortex	(−51, −19, 7)	L—Inferior parietal lobule	(−61, −40, 36)	0.31	0.41	0.26
	R—S2	(48, −10, 16)	L—Inferior parietal lobule	(−61, −40, 36)	0.21	0.23	0.09
	R—Auditory cortex	(55, −12, 6)	L—Inferior parietal lobule	(−61, −40, 36)	0.38	0.4	0.25
SMN-ECN (3)	L—Somato-motor network component A	(−23, −23, 63)	L—Inferior parietal lobule	(−48, −52, 52)	−0.18	−0.01	−0.17
	L—S2	(−50, −15, 17)	L—Inferior parietal lobule	(−48, −52, 52)	−0.12	−0.04	−0.18
	R—Auditory cortex	(55, −12, 6)	L—Inferior parietal lobule	(−48, −52, 52)	−0.07	0.02	−0.11
DAN-ECN	L—Post-central cortex	(−36, −38, 55)	L—Inferior parietal lobule	(−48, −52, 52)	0.15	0.25	0.12
SMN- DMN	L—Somato-motor network component A	(−23, −23, 63)	L—Inferior parietal lobule	(−53, −54, 30)	−0.3	−0.16	−0.32
*RD vs. HC (Component 3)*							
SN – SN (5)	L—Inferior parietal lobule	(−61, −40, 36)	R—Lateral prefrontal cortex	(32, 49, 25)	0.29	0.2	0.39
	L—Inferior parietal lobule	(−61, −40, 36)	R—Ventrolateral prefrontal cortex	(45, 46, 0)	0.29	0.15	0.39
	R—Inferior parietal lobule	(52, −49 49)	R—Ventrolateral prefrontal cortex	(45, 46, 0)	0.34	0.14	0.43
	L—Inferior parietal lobule	(−61, −40, 36)	R—Posterior-medial prefrontal cortex	(6, 28, 33)	0.16	0.02	0.19
	R—Posterior-medial prefrontal cortex	(6, 28, 33)	R—anterior Cingulate cortex	(5, 19, 23)	0.68	0.53	0.69
SN – ECN (4)	R—Dorsal prefrontal cortex	(13, 15, 65)	L—Inferior parietal lobule	(−48, −52, 52)	−0.06	−0.19	−0.01
	R—Ventrolateral prefrontal cortex	(45, 46, 0)	L—Inferior parietal lobule	(−48, −52, 52)	0.36	0.25	0.42
	L—Inferior parietal lobule	(−61, −40, 36)	L—Posterior-medial prefrontal cortex	(−5, 32, 44)	−0.06	−0.17	0
	R—Ventrolateral prefrontal cortex	(45, 46, 0)	R—Inferior parietal lobule	(52, −49, 49)	0.28	0.17	0.37
ECN – ECN (5)	L—Inferior parietal lobule	(−48, −52, 52)	L—Posterior-medial prefrontal cortex	(−5, 32, 44)	0.45	0.4	0.6
	L—Intraparietal sulcus	(−39, −50, 47)	R—Dorsolateral prefrontal cortex	(32, 29, 46)	0.12	−0.03	0.12
	L—Inferior parietal lobule	(−48, −52, 52)	R—Dorsolateral prefrontal cortex	(32, 29, 46)	0.26	0.14	0.37
	R—Inferior parietal lobule	(52, −49 49)	R—Dorsolateral prefrontal cortex	(32, 29, 46)	0.63	0.48	0.72
	L—Inferior parietal lobule	(−48, −52, 52)	R—Posterior-medial prefrontal cortex	(4, 39, 42)	0.08	−0.03	0.15
ECN – DMN (2)	R—Posterior-medial prefrontal cortex	(4, 39, 42)	L—Inferior parietal lobule	(−53, −54, 30)	0.44	0.26	0.44
	L—Inferior parietal lobule	(−48, −52, 52)	R—Dorsal prefrontal cortex	(9, 50, 39)	−0.18	−0.34	−0.18
*FED vs. RD*							
*(Component 4)*							
SMN-DAN	R—Insula	(36, −18, 9)	R—Parieto-occipital cortex	(46, −70, 20)	0	−0.17	−0.15
SMN-DMN (9)	L—S2	(−50, −15, 17)	L—Parahippocampal cortex	(−27, −31, −18)	0.04	−0.17	−0.08
	L—Somato-motor network component A	(−23, −23, 63)	R—Inferior parietal lobule	(49, −70, 30)	−0.2	−0.39	−0.32
	L—S2	(−50, −15, 17)	R—Inferior parietal lobule	(49, −70, 30)	−0.18	−0.36	−0.32
	L—Auditory cortex	(−51, −19, 7)	R—Inferior parietal lobule	(49, −70, 30)	−0.23	−0.42	−0.36
	R—Insula	(36, −18, 9)	R—Inferior parietal lobule	(49, −70, 30)	−0.06	−0.23	−0.23
	R—Auditory cortex	(55, −12, 6)	R—Inferior parietal lobule	(49, −70, 30)	−0.26	−0.44	−0.44
	L—S2	(−50, −15, 17)	R—Parahippocampal cortex	(27, −28, −19)	0.01	−0.17	−0.02
	L—Insula	(−34, −21, 10)	R—Parahippocampal cortex	(27, −28, −19)	0.07	−0.09	0.07
	L—Auditory cortex	(−51, −19, 7)	R—Parahippocampal cortex	(27, −28, −19)	0.03	−0.14	−0.04

A disrupted component with hyperconnectivity and a component with hypoconnectivity were found in the RD group, compared to HCs (*p* < 0.001; Fig. 1b and Table 1). The component with hyperconnectivity (Component 2, including 9 connections) mainly included connectivity of SMN-SN, SMN-ECN, SMN-DMN, and DAN-ECN. Besides, the average FC value of Component 2 in the FED group was also greater than that in the HCs, although the difference was insignificant (RD > FED > HC). The component with hypoconnectivity (Component 3, including 16 connections) included intra-network connectivity of SN and ECN, and inter-network connectivity of SN-ECN. The average FC value of Component 3 in the FED group was lower than that in the HCs, and the difference was also insignificant (RD < FED < HC).

A direct comparison between the FED and RD groups showed a significant component with hypoconnectivity (Component 4, including 10 connections) in the FED group (*p* < 0.001; Fig. 1c and Table 1). This component mainly included inter-network connectivity of SMN-DMN, which covered all the connections in Component 1.

### Abnormal FC in the rFED and rRD groups

**Fig. 2:**
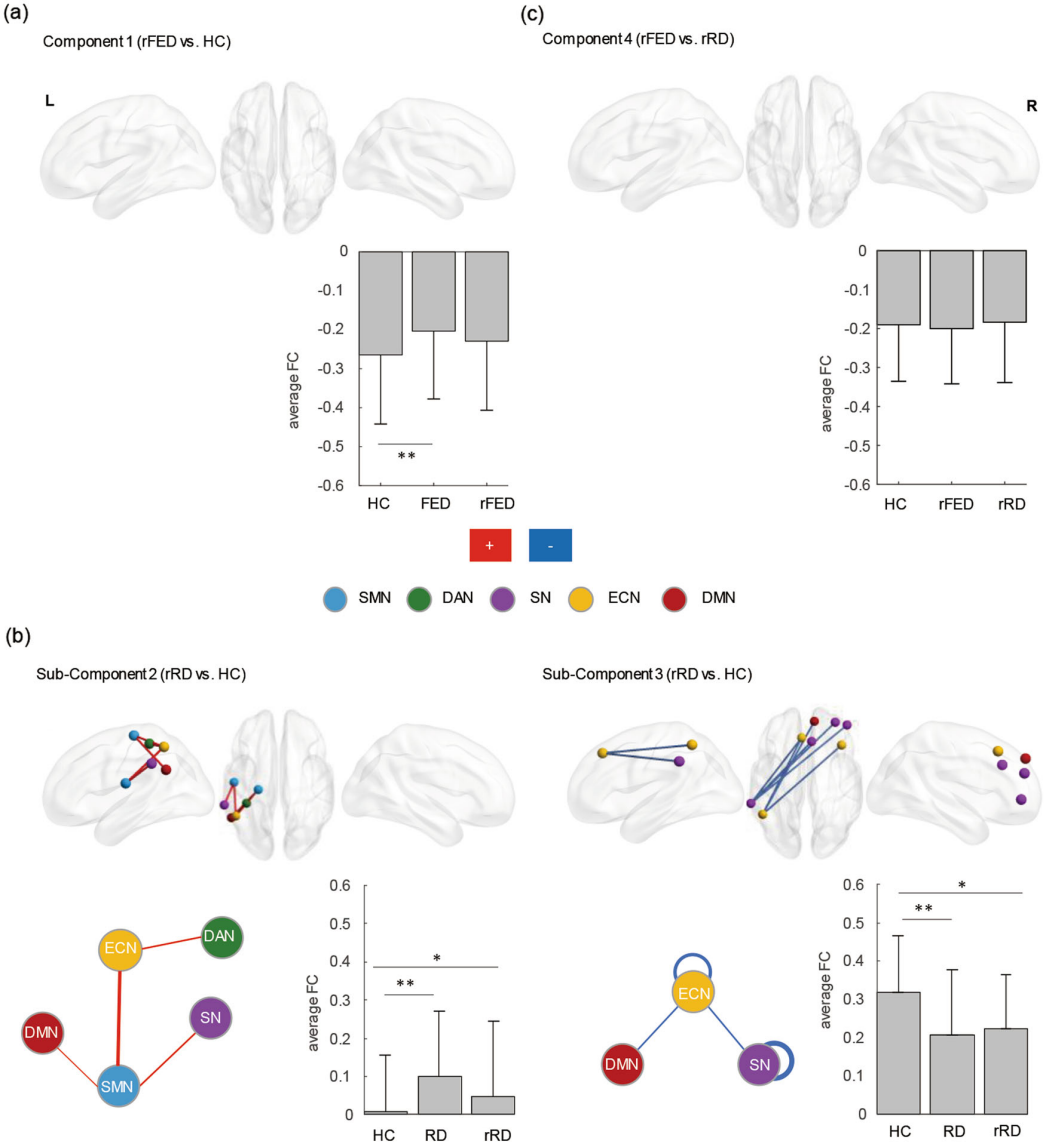
Disrupted components in rFED and rRD. The components were identified using the NBS analysis (the top row). The colors of nodes indicate intrinsic FCN membership as defined by the Yeo parcellation (the bottom row). The thickness of the lines represents the number of significant intra- (loops) and inter-network connections, with thicker lines representing a greater number of significant connections. The blue lines indicate hypoconnectivity in patients with rRD. The red lines indicate hyperconnectivity in patients with rRD. **p < 0.001, *p < 0.05. **a** Aberrant functional connectivity (FC) of Component 1 in patients with rFED, compared to HCs. Bar plots show the average FC value of Component 1 in the FED, rFED and HC groups. **b** Aberrant FC of Component 2 and Component 3 in patients with rRD, compared to HCs, respectively. Bar plots show the average FC value of Component 2 and Component 3 in the FED, rFED and HC groups, respectively. **c** Aberrant FC of Component 4 in patients with rFED, compared to those with rRD. Bar plots show the average FC value of Component 4 in the rFED, rRD and HC groups. SMN: somatomotor network, DAN: dorsal attention network, SN: salience network, ECN: executive control network, DMN: default mode network, L: left, R: right, FED: first-episode depression, RD: recurrent depression, rFED: remitted FED, rRD: remitted RD; HC: healthy control.

There was no significant change before and after treatment in both the rFED and rRD groups. No significant difference was found between the rFED and rRD groups and HCs when the primary threshold was set at 3. When the primary threshold was set at 2, there was no significant difference between the rFED and HC groups (Fig. 2a), while there was a sub-Component 2 with hyperconnectivity (including 5 connections) and a sub-Component 3 with hypoconnectivity (including 7 connections) in the rRD group, compared to HCs (*p* < 0.05; Fig. 2b). The sub-Component 2 included inter-network connectivity of SMN-SN, SMN-ECN, SMN-DMN, and DAN-ECN, and the sub-Component 3 mainly included intra-network connectivity of SN and ECN. However, there was no significant difference between the rFED and rRD groups (Fig. 2c). Detailed information was presented in Table 2.

**Table 2:** Aberrant functional connectivity of sub-component 2 and sub-component 3 in rRD patients, compared to HC.

Significant connections					Functional connectivity		
Network	Seed region	Coordinates (*x*, *y*, *z*)	Target region	Coordinates (*x*, *y*, *z*)	rFED	rRD	HC
*rRD vs. HC (sub-Component 2)*							
SMN-SN (1)	L—S2	(−50, −15, 17)	L—Inferior parietal lobule	(−61, −40, 36)	0.19	0.17	0.12
SMN-ECN (2)	L—Somato-motor network component A	(−23, −23, 63)	L—Inferior parietal lobule	(−48, −52, 52)	−0.03	−0.14	−0.17
	L—S2	(−50, −15, 17)	L—Inferior parietal lobule	(−48, −52, 52)	−0.08	−0.13	−0.18
DAN-ECN	L—Post-central cortex	(−36, −38, 55)	L—Inferior parietal lobule	(−48, −52, 52)	0.22	0.19	0.12
SMN- DMN	L—Somato-motor network component A	(−23, −23, 63)	L—Inferior parietal lobule	(−53, −54, 30)	−0.23	−0.32	−0.32
*rRD vs. HC (sub-Component 3)*							
SN – SN (3)	L—Inferior parietal lobule	(−61, −40, 36)	R—Lateral prefrontal cortex	(32, 49, 25)	0.3	0.27	0.39
	L—Inferior parietal lobule	(−61, −40, 36)	R—Ventrolateral prefrontal cortex	(45, 46, 0)	0.15	0.23	0.39
	L—Inferior parietal lobule	(−61, −40, 36)	R—Posterior-medial prefrontal cortex	(6, 28, 33)	0.04	0.11	0.19
SN – ECN	L—Inferior parietal lobule	(−61, −40, 36)	L—Posterior-medial prefrontal cortex	(−5, 32, 44)	−0.14	−0.11	0
ECN – ECN (2)	L—Inferior parietal lobule	(−48, −52, 52)	L—Posterior-medial prefrontal cortex	(−5, 32, 44)	0.19	0.23	0.6
	L—Inferior parietal lobule	(−48, −52, 52)	R—Posterior-medial prefrontal cortex	(4, 39, 42)	0.22	0.27	0.15
ECN – DMN	L—Inferior parietal lobule	(−48, −52, 52)	R—Dorsal prefrontal cortex	(9, 50, 39)	−0.33	−0.18	−0.18

### Correlation analysis in pooled patients

**Fig. 3:**
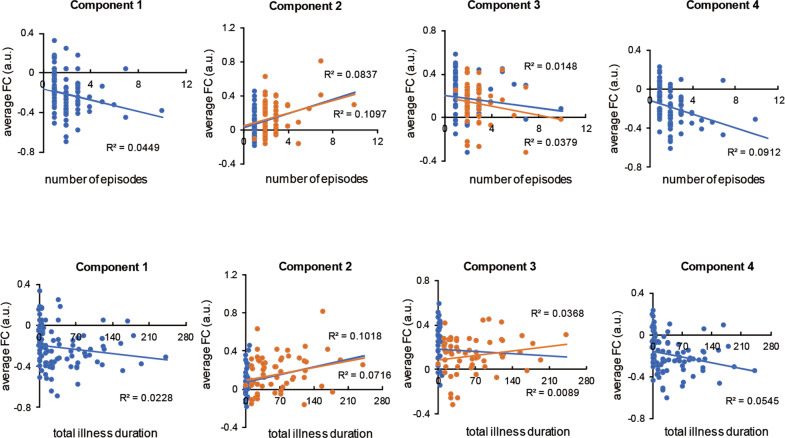
The correlation between the average FC value of disrupted components and the number of episodes and total illness duration in MDD patients. The blue dots represent all the MDD (FED and RD) patients. The orange dots represent the RD patients.

The average FC value of the Component 1 was negatively correlated with the number of episodes (*r* = −0.212, *p* = 0.039) in patients with MDD. The average FC value of the Component 2 was positively correlated with the number of episodes (*r* = 0.331, *p* = 0.001; *r* = 0.289, *p* = 0.025) and the total duration of illness (*r* = 0.319, *p* = 0.002; *r* = 0.268, *p* = 0.039) in both the MDD and RD groups. Besides, the average FC value of the Component 4 was negatively correlated with the number of episodes (*r* = −0.302, *p* = 0.003) and the total duration of illness (*r* = −0.233, *p* = 0.023) in patients with MDD. The correlation results were shown in Fig. 3.

## Discussion

Using a connectome-wide analysis, we conducted a longitudinal study across different phases of MDD to investigate the progressive nature of intrinsic FC in patients with this disorder. Our results provided two pieces of evidence confirming the progressive nature of intrinsic FC: RD showed more extensive and severe FC abnormalities compared to FED, and some of the abnormalities would remain even in the remission phase of RD. Additionally, the hypoconnectivity in FED and hyperconnectivity in RD were associated with the number of episodes and the total illness duration. The first piece of evidence not only revealed the differences in FC between FED and RD across different phases, but also provided an insight for the progressive process that the improvement of FC was behind the relief of depression symptoms after a depressive episode. The second piece of evidence indicated that the brain functional alteration might deteriorate with an increasing number of episodes. The two pieces of evidence were consistent and provided substantial support for the identification of the progressive nature of intrinsic FC in MDD.

The result showed group, compared more severe FC disruption and extensive FCN abnormality in RD than FED, which was in line with the previous studies directly comparing the clinical symptoms between FED and RD. The FC aberrance of Component 2 found in RD was inter-network FC, involving the networks from SMN to DMN, SN and ECN. As revealed by previous studies, the aberrant inter-network FC could be interpreted as the neural underpinnings of the pervasive influence of psychomotor retardation on internal mentation, emotional processes and cognitive control^36^. In addition, Component 2 in RD was found positively correlated with the number of episodes and the total illness duration in the present study. These findings were in line with the previous studies which showed that psychomotor retardation was progressively deteriorated with accumulating depressive episodes^37^, providing a further explanation for the persistent psychomotor retardation as a primary deficit that might lead to other functional impairments^38^. Likewise, aberrant connections of Component 3 found in RD concentrated in SN and ECN, involving both inter-network and intra-network FC. SN and ECN were considered as the neural underpinnings of emotion processing and cognitive control processing, respectively, while the inter-network FC from SN to ECN might be the foundation of the interaction between emotion and cognitive processing^39^. This aberrant component would be the neural underpinnings of more prominent and extensive affective and cognitive deficits that were presented in RD rather than FED, which was consistent with the characteristics of chronic and recurrent MDD, such as persistent negative mood, biased cognitive style and progressive neurocognitive deficits^2,40,41^.

The results of our investigation of the FC alterations across different phases of MDD support the view that MDD is a chronic disease characterized by progressive functional impairments. Previous studies have revealed that some prominent symptoms and aberrant FC shown in acute depressive episodes would remain even in the clinical remission phase^42–44^. And these residual symptoms and abnormalities might lead to functional impairment, recurrence and poor prognosis of MDD^4,45,46^. Our results showed that the FC aberrance in the episode phase of both FED and RD did not improve significantly even the patient had achieved remission, indicating that even though the depressive symptoms have relieved, the FC abnormality could not fully recover to a normal level. The improvement of FC abnormality lagged behind other depression-related symptoms in time and degree, meaning that the abnormalities might need more time to recover or might not be able to fully recover to normal levels at all. In addition, no significant difference was found in FC aberrance between remitted FED patients and HCs, while remitted RD patients still had a component with hypoconnectivity and a component with hyperconnectivity. This result indicates that the abnormalities in RD are more difficult to reverse than that in FED, and are more likely to accumulate and progressively deteriorate with an increasing number of episodes. These findings have provided a further explanation for the more serious impairment in RD, as well as further evidence for the progressive nature of intrinsic FC in patients with MDD.

Notably, except for the chronic and progressive functional network abnormalities in MDD, we could not rule out the possibility that Component 3 in RD tends to be a trait-like characteristic because no significant correlation was found between Component 3 and the number of episodes and total illness duration. Besides, the average FC of Component 3 did not alter significantly after 6 months of treatment. Component 3 mainly concentrated in SN and ECN, which are the core networks involving in emotional and cognitive processing. From the perspective of behavioral performance, subjects who are susceptible to depression and more likely to have recurrent episodes often show more obvious negative cognitive biases, more negative coping styles and poorer neurocognitive functions^41,47,48^, leading to poor emotional and cognitive control processing. Therefore, this component might be the neural underpinning of depression susceptibility. However, the reason might also be the non-linear characteristics of the alteration of this component.

Another interesting finding of the present study was that the aberrant component in FED seemed to be a temporary adaptive response to stress. Stress has been confirmed as one of the major causes of MDD^49^, with around 70% of MDD patients having dysfunction of the HPA axis, which leads to ineffective coping with acute and chronic stress^50^. Studies have found that alterations of neuropsychological, neuroimmune and neuroendocrine systems are involved in the process of stress adaptation^51–53^. Resting-state MRI studies also reported that alterations of medial corticolimbic circuits might be a potential target of stress adaptation^54^. The Component 1 aberrance was significant in FED but not significant in RD, and the average FC value of Component 1 in RD was almost the same as that in HCs. Although there was no significant difference in this aberrant component between the acute episode and the remission phase, this component still improved to some extent when the MDD patients achieved remission (*p* > 0.05, compared with the HCs). Accordingly, the abnormality of this component was negatively correlated with the number of episodes and the total illness duration in pooled MDD patients; this implies that this aberrant component might gradually return to the HC level with the increasing number of episodes. These results support the view that the aberrant component in FED might be a protective response to stress. In addition, this aberrance might also be a compensatory change of networks^55^.

Although the present study was strengthened by the longitudinal design, several limitations should still be noted. Firstly, some patients were more likely to consider first-class hospitals in major cities (e.g., Zhengzhou or Beijing) and withdrew from the study due to poor response to paroxetine, resulting in a significantly smaller number of unremitted patients. This precluded us from making direct comparisons between remitted and unremitted patients, although there were no significant differences in demographic/clinical features and the FC at baseline between remitted patients and other participants (i.e., unremitted patients and dropouts). Secondly, the direct evidence for the neuroprogressive nature of MDD should come from the significant change shown by repeated measures of FC in MDD patients (MRI scans in each progressive episode of the same MDD patients), however, we were precluded from making such a comparison due to the mismatch of the sample size of the remitted and unremitted groups. Therefore, we conducted correlation analyses, which showed more extensive and severe FC abnormalities in FD, compared to FED, and the abnormalities were more difficult to reverse in patients with RD. This result also suggested that the brain functional abnormalities are more likely to accumulate and progressively deteriorate with an increasing number of episodes. Thirdly, the evidence found in our study was only of moderate strength in support of the coexistence of progressive, trait-like and temporary adaptive FC alterations in MDD. Future researches, potentially utilizing a longitudinal design and including more detailed information about subsequent episodes and remission phases, could explore this topic.

In summary, the present study might be the first one to investigate FC changes across different phases of MDD through a connectome-wide analysis in a relatively large cohort. The results provided new evidence supporting the progressive nature of intrinsic FC abnormality across the course of depression. The results also provided moderate evidence supporting the coexistence of progressive, trait-like and temporary adaptive FC alterations in MDD. These findings allow us to gain an insight into the underlying connectome mechanism of the progressive nature of MDD that calls for full course management.

## Supplementary information

Supplementary Information

Supplementary Figures

Supplementary Tables
