# Supplementary material for: The neuroprogressive nature of major depressive disorder: evidence from an intrinsic connectome analysis
Source: Transl Psychiatry. 2021 Feb 4;11:102. doi: 10.1038/s41398-021-01227-8 (PMC7862649; doi:10.1038/s41398-021-01227-8)
Supplement: Supplementary file 1 — Supplementary Information [file 41398_2021_1227_MOESM1_ESM.docx]

**Supplementary Information**

**1. Inclusion and exclusion criteria**

From 2013 to 2018, 107 major depressive disorder (MDD) patients and 117 healthy controls (HCs) were recruited from Zhumadian Psychiatric Hospital (Henan, China) and its surrounding communities. The common inclusion criteria of the two groups were: 1) 18 ~ 55 years old; 2) education ≥ 6 years; 3) right-handed. For the MDD group, the additional inclusion criteria were: 1) diagnosed with MDD by two attending psychiatrists using Structured Clinical Interview for DSM-IV (SCID-IV); 2) currently suffering from at least moderate depression, with a score of ≥ 20 on the 24-item Hamilton Depression Rating Scale (HAM-D_24_); 3) not taking psychotropic drugs at least 2 weeks (6 weeks for fluoxetine) before inclusion. Other inclusion criteria for HCs were: 1) no current or lifetime diagnosis of psychiatric disorder or substance-related disorders; 2) HAMD_24_ ≤ 7; 3) no history of psychotropic substance or drug use. The exclusion criteria for both groups were: 1) any other DSM-IV psychiatric disorder or major physical illness diagnosed at present or lifetime; 2) history of head injury or loss of consciousness for more than 5 minutes; 3) DSM-IV alcohol or drug dependence; 4) pregnancy or breastfeeding; 5) color-blindness; 6) abnormal urine toxicology or thyroid screening results.

**2. MRI data acquisition, preprocessing and functional connectivity network (FCN) construction**

**2.1 MRI Data Acquisition**

All subjects were scanned using a 3T MR scanner (Signa HDxt MR, GE Healthcare, Milwaukee, WI). During scanning, foam pads were used to reduce head motion and scanner noise. Functional images were obtained using a gradient (GRE) echo-planar imaging sequence with axial scanning according to the following parameters: repetition time (TR) =2,000 ms; echo time (TE) = 30 ms; field of view (FOV) = 220 × 220 mm^2^; flip angle = 90^o^; slice thickness = 4 mm; slice gap = 0.6 mm; matrix size = 64 × 64; slices = 33; A 6-minute (180 time points) fMRI scan was collected for each subject. We also acquired high-resolution 3D brain anatomical images using a T1-weighted BRAVO sequence according to the following parameters: TR = 6.8 ms, TE = 2.5 ms, flip angle = 9^o^, slice gap = 0 mm, turnover time (TI) = 1100 ms, NEX = 1, FOV = 256 × 256 mm^2^, matrix size= 256 × 256, and 192 contiguous sagittal slices were collected with slice thickness = 1mm.

**2.2 Data preprocessing**

For the functional imaging data of each subject, the first 10 time points were discarded to remove possible T1 stabilization effects. Next, imaging data were preprocessed using the statistical parametric mapping software package SPM12 (http://www.fil.ion.ucl.ac.uk/spm). Preprocessing steps mainly included the removal of sudden spikes caused by significant head motion, slicing time correction, spatial realignment correction, normalization, spatial smoothing, and temporal band-pass filtering ^1, 2^. Slicing time correction was utilized to compensate for slice acquisition dependent time shifts of each volume. Six rigid body translation and rotation parameters were applied to correct head motion. It should be noted that the subjects which included had less than 2 mm translation and 2° of rotation in any of the x-, y-, and z-axes. Normalization included rs-fMRI data written into the Montreal Neurological Institute (MNI) space at a 3 × 3 × 3mm^3^ resolution using the nonlinear transformation calculated on the corresponding anatomic images. The normalized functional volumes were spatially smoothed using a 6-mm full width at half maximum (FWHM) Gaussian filter kernel. Briefly, the linear trend over each scan was removed and a temporal bandpass filter from 0.01 to 0.08 Hz was performed. Prior to spatial smoothing, to reduce hardware noise, the draining vessel effect, and motion artifacts on each voxel of gray matter, we further regressed the following nuisance variables: six rigid body motion parameters, three mean signals from the white matter (WM), cerebrospinal ﬂuid (CSF) and whole-brain global signals, as well as their first-order derivative terms. The residuals from the functional data were used for further analysis. After data preprocessing, 5 MDD patients and 6 HC were excluded for excessive head motions. Considering that resting-state FC can be affected by micromovements from volume to volume ^1, 3^, we calculated the frame-wise displacement (FD) values of each subject and estimated the differences of FD across first-episode MDD (FED), recurrent MDD (RD), and HC groups; and remitted FED (rFED), remitted RD (rRD), and HC groups. No significant differences were found in these groups.

**2.3 Construction of FCN**

A whole-brain FCN was constructed for each subject. The FCN comprised nodes based on a previously established functional parcellation of the human cerebral cortex and the human striatum ^4, 5^. This parcellation was derived by clustering the whole-brain FCN of 500 subjects (along with a 500 subject replication cohort) according to the similarity of regions’ functional connectivity (FC) profiles. This procedure resulted in seven clusters, whose boundaries shared a close correspondence to the known topographic boundaries of visual network (VN), somatomotor (SMN), Limbic, executive control (ECN), dorsal attention (DAN), salience (SN) and default mode (DMN) networks. Detailed visualization of each network can be seen in Supplementary Figure S1. Across all the 7 sub-networks, 132 separated anatomical regions of interest (ROIs) were included. These ROIs were then used to represent nodes in FCNs. The functional connection between nodes *i* and *j* was defined as the Fisher-z transformed Pearson product-moment correlation of the representative BOLD (Blood Oxygen Level-Dependent) time series recorded at those nodes. The representative BOLD time series of a ROI was obtained by averaging the time series of all the voxels in this ROI. The Pearson’s correlation coefficients were then calculated between each pair of ROIs. To improve the normality of the correlation coefficients, Fisher’s r-to-z transform was performed to convert the correlation coefficients to z-values.

**2.4** **Network-based Statistics (NBS) analysis**

The NBS incorporated graph model to identify the significance of any connected components evident at FED and RD compared to HC ^6^. NBS was implemented as follows: The first step is to independently test the hypothesis of interest at every connection with an appropriate statistical test. Each connection is therefore endowed with a single test statistic value quantifying the evidence in favor of the null hypothesis. The second step is to choose a test statistic threshold, referred to as the primary threshold. The connections with a test statistic value exceeding this threshold are admitted to a set of supra-threshold connections. Connections comprising this set represent potential candidates for which the null hypothesis can be rejected. The third step is to identify topological clusters among the set of supra-threshold connections with a breadth or depth search. In this context, a connected graph component is a set of supra-threshold connections for which a path can be found between any two nodes.

The final step is to compute an FWER-corrected *p*-value for each component using permutation testing. The basic premise of permutation testing is that the correspondence between data points and their labels can be randomly rearranged under the null hypothesis without affecting the test statistic. For each permutation, the first three steps of the NBS are repeated on the permuted data. In particular, the hypothesis of interest is tested at every connection using the same statistical test, a set of supra-threshold connections is defined using the same threshold and any connected graph components are then identified. The size of the largest component is recorded for each permutation, thereby yielding an empirical null distribution for the size of the largest component size.

The one-sided FWER-corrected *p*-value for a component of the given size is then estimated as the proportion of permutations for which the largest component was of the same size or greater.
