# Supplementary material for: The neuroprogressive nature of major depressive disorder: evidence from an intrinsic connectome analysis
Source: Transl Psychiatry. 2021 Feb 4;11:102. doi: 10.1038/s41398-021-01227-8 (PMC7862649; doi:10.1038/s41398-021-01227-8)
Supplement: Supplementary file 2 — Supplementary Figures [file 41398_2021_1227_MOESM2_ESM.docx]

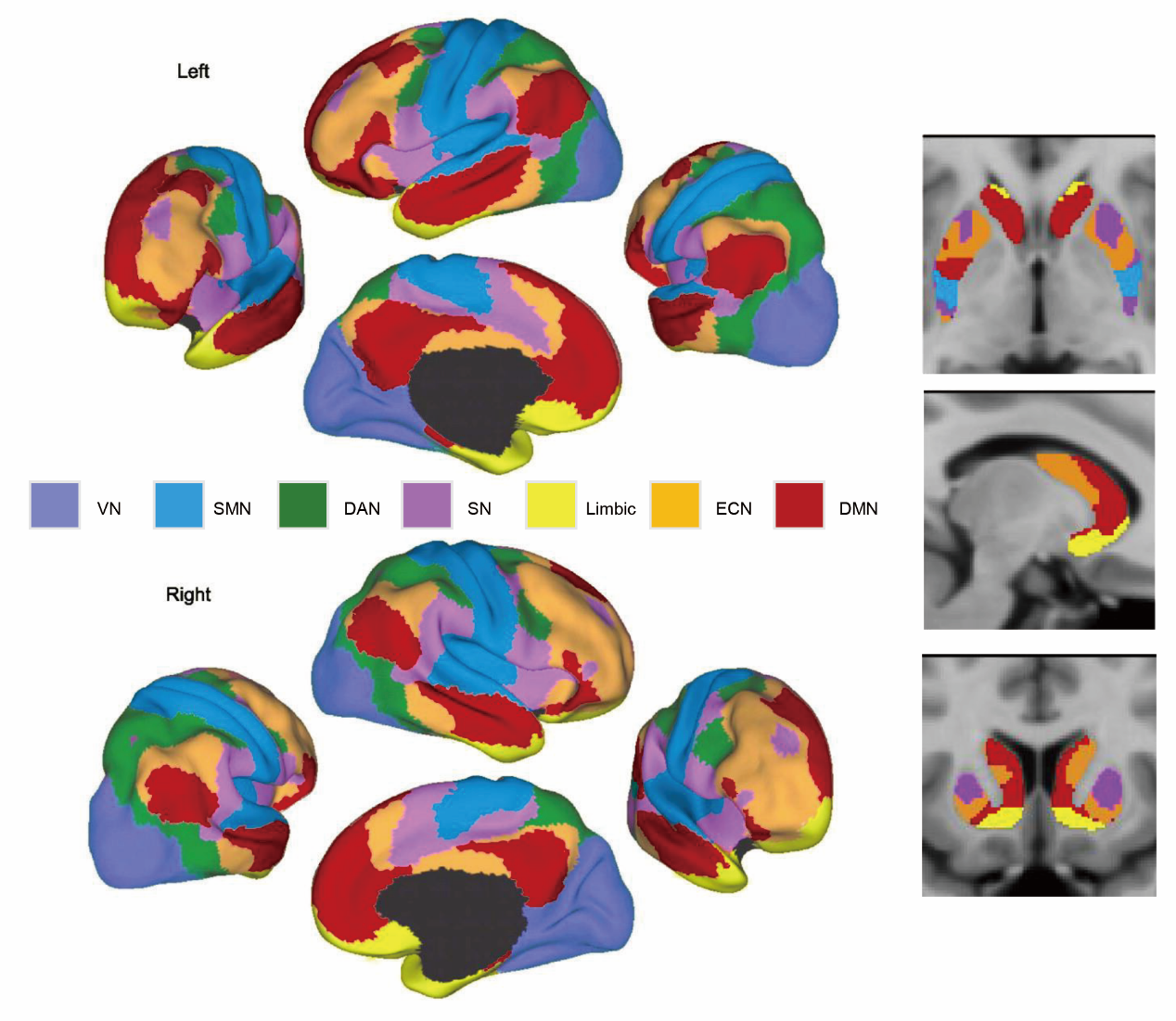


**Supplementary Figure S1. Surface rendering of all seven resting-state functional connectivity networks comprising 132 brain regions (114 cortical regions and 18 sub-regions of striatum) in total on both left hemisphere (left) and right hemisphere (right).** The inflated surfaces are provided by Caret. The color map indicates colors picturing each of the seven networks and their regions on the surfaces. VN: visual network, SMN: somatomotor network, DAN: dorsal attention network, SN: salience network, ECN: executive control network, Limbic: limbic, DMN: default mode network.


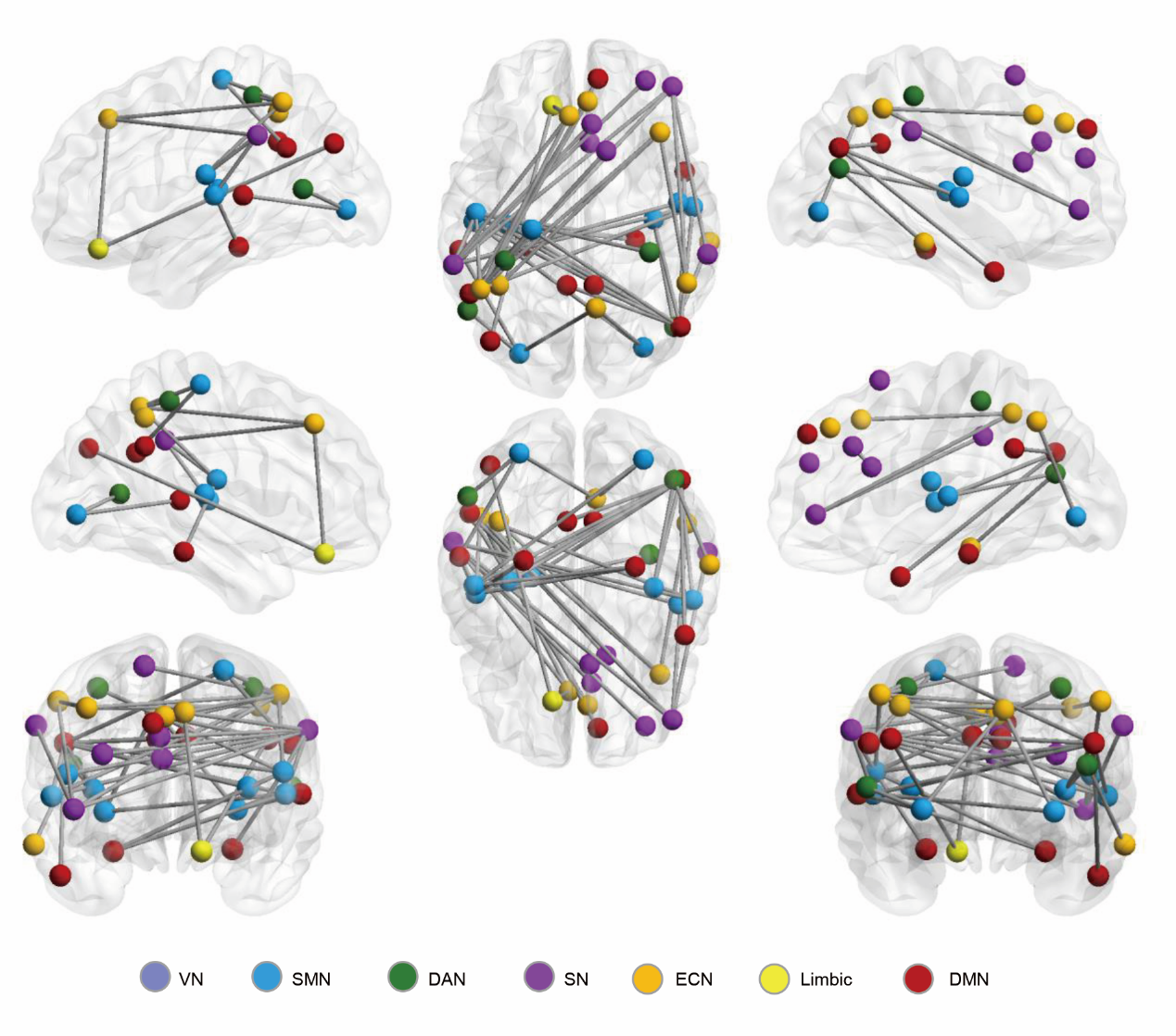


**Supplementary Figure S2. Significant functional connectivity across FED, RD, and HC.** VN: visual network, SMN: somatomotor network, DAN: dorsal attention network, SN: salience network, ECN: executive control network, Limbic: limbic, DMN: default mode network, FED: first-episode depression, RD: recurrent depression, HC: healthy control.
