# Supplementary material for: The neuroprogressive nature of major depressive disorder: evidence from an intrinsic connectome analysis
Source: Transl Psychiatry. 2021 Feb 4;11:102. doi: 10.1038/s41398-021-01227-8 (PMC7862649; doi:10.1038/s41398-021-01227-8)
Supplement: Supplementary file 3 — Supplementary Tables [file 41398_2021_1227_MOESM3_ESM.docx]

Supplementary Table S1. The demographic and clinical characteristics of the MDD, rMDD, and HC.

| Characteristics | MDD  (mean ± s.d.) | | HC  (mean ± s.d.)  (*n* = 111) | rMDD  (mean ± s.d.) | | *p_1_* | *p_2_* |
| --- | --- | --- | --- | --- | --- | --- | --- |
|  | FED  (*n* = 35) | RD  (*n* = 60) |  | rFED  (*n* = 20) | rRD  (*n* = 36) |  |  |
| ^a^Age (years) | 32.11 ± 8.42 | 34.82 ± 8.84 | 34.85 ± 8.90 | 32.85 ± 9.33 | 36.44 ± 9.20 | 0.25 | 0.35 |
| ^b^ Gender (F/M) | 17/18 | 38/22 | 59/52 | 9/11 | 23/13 | 0.30 | 0.35 |
| ^a^ Education (years) | 11.23 ± 3.47 | 10.25 ± 3.34 | 10.78 ± 3.24 | 11.45 ± 3.59 | 10.44 ± 3.42 | 0.34 | 0.56 |
| ^c^ HAM-D_24_ | 34.89 ± 7.47 | 32.40 ± 6.80 | -- | 2.71 ± 2.02 | 3 ± 2.20 | 0.11 | 0.64 |
| ^c^ Total illness duration (months) | 3.63 ± 2.22 | 66.13 ± 53.75 | -- | 6.73 ± 2.13 | 70.94 ± 59.44 | < 0.001 | < 0.001 |
| ^c^ Number of episodes | 1 ± 0 | 2.83 ± 1.52 | -- | 1 ± 0 | 2.77 ± 1.68 | < 0.001 | < 0.001 |

MDD = MDD patients with current episode; FED = first-episode depression patients, RD = recurrent depression patients, HC = healthy controls, rMDD = remitted MDD patients.

s.d. = standard deviation.

^a^ANOVA, ^b^Chi-test, ^c^Two-sample t-tests

*p_1_* values denote the statistical result across FED, RD, and HC.

*p_2_* values denote the statistical result across rFED, rRD, and HC.

Supplementary Table S2. Significant functional connectivity across FED, RD, and HC.

| Significant connections |  |  |  |  |
| --- | --- | --- | --- | --- |
| Network | Seed region | Coordinates (x, y, z) | Target region | Coordinates (x, y, z) |
| VN - SMN | R - Extrastriate cortex | （31, -80, 1） | L – Auditory cortex | (-51, -19, 7) |
| VN – DAN | L - Extrastriate cortex | （-29, -83, 0） | L - Temporo-occipital cortex | (-54, -62 10) |
| VN – ECN (2) | L - Extrastriate cortex | （-29, -83, 0） | R - Precuneus | (8, -61, 45) |
|  | R - Extrastriate cortex | （31, -80, 1） | R - Precuneus | (8, -61, 45) |
| VN - DMN | L - Extrastriate cortex | （-29, -83, 0） | L - Temporo-parietal cortex | (-58, -33 7) |
| SMN - DAN (2) | R - Insula | (36, -18, 9) | R – Parieto-occipital cortex | (46, -70, 20) |
| SMN - SN (4) | L - S2 | (-50, -15, 17) | L – Inferior parietal lobule | (-61, -40, 36) |
|  | L – Auditory cortex | (-51, -19, 7) | L – Inferior parietal lobule | (-61, -40, 36) |
|  | R - S2 | (48, -10, 16) | L – Inferior parietal lobule | (-61, -40, 36) |
|  | R – Auditory cortex | (55, -12, 6) | L – Inferior parietal lobule | (-61, -40, 36) |
| SMN - ECN (3) | L - Somato-motor network component A | (-23, -23, 63) | L – Inferior parietal lobule | (-48, -52, 52) |
|  | L - S2 | (-50, -15, 17) | L – Inferior parietal lobule | (-48, -52, 52) |
|  | R - Auditory cortex | (55, -12, 6) | L – Inferior parietal lobule | (-48, -52, 52) |

| Table S2 (continued) | | | | | |
| --- | --- | --- | --- | --- | --- |
| Significant connections |  |  | |  |  |
| Network | Seed region | Coordinates (x, y, z) | | Target region | Coordinates (x, y, z) |
| SMN - DMN (11) | L - S2 | | (-50, -15, 17) | R – Inferior parietal lobule | (49, -70, 30) |
|  | L – Insula | | (-34, -21, 10) | R – Inferior parietal lobule | (49, -70, 30) |
|  | R - Insula | | (36, -18, 9) | R – Inferior parietal lobule | (49, -70, 30) |
|  | R – Auditory cortex | | (55, -12, 6) | R – Inferior parietal lobule | (49, -70, 30) |
|  | L - Somato-motor network component A | | (-23, -23, 63) | L – Inferior parietal lobule | (-53, -54, 30) |
|  | L - S2 | | (-50, -15, 17) | L - Parahippocampal cortex | (-27, -31, -18) |
|  | L - Somato-motor network component A | | (-23, -23, 63) | R - Inferior parietal lobule | (49, -70, 30) |
|  | L – Auditory cortex | | (-51, -19, 7) | R - Inferior parietal lobule | (49, -70, 30) |
|  | L – S2 | | (-50, -15, 17) | R - Parahippocampal cortex | (27, -28, -19) |
|  | L – Insula | | (-34, -21, 10) | R - Parahippocampal cortex | (27, -28, -19) |
|  | L – Auditory cortex | | (-51, -19, 7) | R - Parahippocampal cortex | (27, -28, -19) |
| DAN - ECN (2) | L – Post-central cortex | | (-36, -38, 55) | L – Inferior parietal lobule | (-48, -52, 52) |
|  | R –Parieto-occipital cortex | | (46, -70, 20) | R – Temporal cortex | (63, -29, -16) |
| SN – SN (5) | L – Inferior parietal lobule | | (-61, -40, 36) | R - Lateral prefrontal cortex | (32, 49, 25) |
|  | L – Inferior parietal lobule | | (-61, -40, 36) | R - Ventrolateral prefrontal cortex | (45, 46, 0) |
|  | R – Inferior parietal lobule | | (62, -35, 38) | R - Ventrolateral prefrontal cortex | (45, 46, 0) |
|  | L – Inferior parietal lobule | | (-61, -40, 36) | R - Posterior-medial prefrontal cortex | (6, 28, 33) |
|  | R - Posterior-medial prefrontal cortex | | (6, 28, 33) | R - anterior Cingulate cortex | (5, 19, 23) |
| Table S2 (continued) | | | | | |
| Significant connections |  |  | |  |  |
| Network | Seed region | Coordinates (x, y, z) | | Target region | Coordinates (x, y, z) |
| SN – ECN (4) | R - Dorsal prefrontal cortex | (13, 15, 65) | | L - Inferior parietal lobule | (-48, -52, 52) |
|  | R - Ventrolateral prefrontal cortex | (45, 46, 0) | | L - Inferior parietal lobule | (-48, -52, 52) |
|  | L - Inferior parietal lobule | (-61, -40, 36) | | L - Posterior-medial prefrontal cortex | (-5, 32, 44) |
|  | R - Ventrolateral prefrontal cortex | (45, 46, 0) | | R - Inferior parietal lobule | (52, -49, 49) |
| ECN – ECN (5) | L - Inferior parietal lobule | (-48, -52, 52) | | L - Posterior-medial prefrontal cortex | (-5, 32, 44) |
|  | L - Intraparietal sulcus | (-39, -50, 47) | | R - Dorsolateral prefrontal cortex | (32, 29, 46) |
|  | L- Inferior parietal lobule | (-48, -52, 52) | | R - Dorsolateral prefrontal cortex | (32, 29, 46) |
|  | R - Inferior parietal lobule | (52, -49 49) | | R - Dorsolateral prefrontal cortex | (32, 29, 46) |
|  | L - Inferior parietal lobule | (-48, -52, 52) | | R - Posterior-medial prefrontal cortex | (4, 39, 42) |
| ECN – DMN (2) | R - Posterior-medial prefrontal cortex | (4, 39, 42) | | L - Inferior parietal lobule | (-53, -54, 30) |
|  | L- Inferior parietal lobule | (-48, -52, 52) | | R - Dorsal prefrontal cortex | (9, 50, 39) |
| Limbic - ECN | L - Orbitofrontal cortex | (-13, 37, -19) | | L - Posterior-medial prefrontal cortex | (-5, 32, 44) |
| Limbic - DMN | L – Orbitofrontal cortex | (-13, 37, -19) | | L - Inferior parietal lobule | (-43, -77, 32) |
| DMN – DMN (3) | L - Posterior cingulate cortex | (-6 -50, 33) | | R - Inferior parietal lobule | (49, -70, 30) |
|  | R - Posterior cingulate cortex | (7, -50, 32) | | R - Inferior parietal lobule | (49, -70, 30) |
|  | R - Anterior temporal cortex | (51, 5, -30) | | R - Inferior parietal lobule | (49, -70, 30) |
